# Linguistic experience and processing speed differentially affect lexical retrieval and structural assembly during language production

**DOI:** 10.3758/s13421-026-01861-x

**Published:** 2026-03-02

**Authors:** Florian Hintz, Mohammad Momenian

**Affiliations:** 1 Research Center Deutscher Sprachatlas, Marburg University, Pilgrimstein 16, 35032 Marburg, Hesse, Germany; 2 Max Planck Institute for Psycholinguistics, Psychology of Language, Nijmegen, The Netherlands; 3 Center for Mind, Brain and Behavior, Marburg University & Justus Liebig University, Marburg, Germany; 4 Department of Linguistics, Faculty of Arts, The University of Hong Kong, Hong Kong SAR, China

**Keywords:** Individual differences, Language production, Processing speed

## Abstract

Our ability to produce words and sentences relies on lexical retrieval and structural assembly processes, which are supported by domain-general skills. In the present study, we adopted an individual-differences approach to examine how linguistic experience and domain-general skills jointly contribute to language production at different levels of complexity. Our participants (*n* = 169) completed three production tasks that capitalized on lexical retrieval (picture naming) and structural assembly (phrase generation and sentence generation) processes, respectively. In addition, they completed 15 tests measuring linguistic experience, processing speed, working memory, and nonverbal reasoning. Our analyses of speech onset latencies and speech durations showed that linguistic experience was primarily associated with faster speech initiation, particularly in picture naming, consistent with its role in supporting efficient lexical access. In contrast, processing speed emerged as a robust predictor of both earlier speech initiation and shorter utterance durations, with effects that were disproportionately larger for phrase and sentence production than for picture naming, highlighting its role in coordinating multiword structural assembly under time pressure. Working memory was linked to a redistribution of planning across time, with higher capacity associated with longer onset latencies but shorter speech durations. By integrating multiple predictors, our study provides novel insights into the cognitive architecture underlying language production and highlights the value of a holistic individual-differences approach.

**Supplementary Information:**

The online version contains supplementary material available at 10.3758/s13421-026-01861-x.

## Introduction

Language production reflects the act of turning a communicative intention into a spoken, written, or signed utterance. In order to speak, language users must retrieve lexical items from long-term memory and—in case of multiword utterances—must engage in morpho-syntactic structure building (Levelt, 1989; for reviews, see Bock & Ferreira, 2014; Vigliocco & Hartsuiker, 2002). Although language production is typically fast and successful, speakers differ markedly in how they produce language (e.g., Unsworth et al., 2011). Such individual variability is evident, among others, in how quickly speakers initiate an utterance and how long they take to complete it. Short speech onset latencies and short utterance durations are commonly taken to reflect efficient lexical retrieval (Meyer et al., 1998) and efficient assembly of lexical items into morpho-syntactic structures (Garrett, 1982). Understanding the sources of this variability provides a valuable window into the cognitive architecture of language production (Kidd et al., 2018).

A central theoretical question concerns how the two core processes underlying production—lexical retrieval and structural assembly—are related. Lexicalist accounts propose that syntactic information is activated as part of lexical retrieval, such that access to individual words drives the selection of syntactic structure (Bock, 1982; Pickering & Branigan, 1998; Tomasello, 2000). In contrast, abstract structural accounts posit that speakers select and generate syntactic structures independently of specific lexical items, guided by message-level constraints (Bock, 1986; Chang et al., 2006; Frazier, 1987; Goldberg, 2005). Hybrid accounts occupy an intermediate position, proposing that production beyond the word level relies on abstract structural representations, but that lexical accessibility can modulate structural planning under certain circumstances (Allum & Wheeldon, 2007; Konopka, 2012; Smith & Wheeldon, 1999). These accounts differ not only in their representational assumptions but also in their implications for how lexical retrieval and structural assembly unfold in time and how they may be differentially supported by cognitive resources.

Language production is also inherently incremental. Speakers need not plan an entire utterance before initiating speech; instead, they may plan in relatively small units and continue planning during articulation (Levelt, 1989; Wheeldon, 2011). Importantly, speakers differ in the degree to which they engage in advance planning, and such differences can reflect strategic trade-offs between initiating speech quickly and planning more of the utterance before speaking (e.g., Swets et al., 2014). As a result, different behavioral measures may index different stages of production. Speech onset latencies are commonly assumed to reflect preparatory processes prior to articulation, including lexical retrieval and early syntactic planning (Treiman et al., 2003), whereas speech durations can capture planning, execution and verbal self-monitoring demands that arise later in the utterance. Examining individual differences in these measures therefore offers a means of probing how planning processes are distributed over time and how they vary across speakers.

A growing body of research suggests that language production is supported not only by domain-specific linguistic but also by domain-general skills (e.g., Cahana-Amitay & Albert, 2014; Federmeier et al., 2020). One such skill is working memory (WM). Studies of both healthy speakers and individuals with language impairments have demonstrated that WM plays an important role in language production, particularly when utterances are structurally complex (Acheson & MacDonald, 2009; Martin & Slevc, 2014; Martin et al., 2014). In models of incremental production, including Levelt’s (1989, 1999) “blueprint of the speaker” and subsequent accounts (e.g., Slevc, 2011; Wheeldon, 2011), WM is assumed to support the temporary maintenance and integration of lexical items and morpho-syntactic frames. As utterance complexity increases, the demands placed on WM likewise increase, as more information must be maintained and coordinated. Connectionist models of production further suggest that WM-related resources are crucial for resolving competition among alternative lexical and structural representations during planning (Chang et al., 2006). Importantly, WM may not simply determine how much information can be planned in advance, but also how flexibly speakers distribute planning across time, potentially leading to differences in onset latencies and speech durations (Swets et al., 2014).

Language production unfolds under substantial temporal pressure. In conversational settings, for example, speakers typically begin responding within a few hundred milliseconds after a turn ends (Heldner & Edlund, 2010). Processing speed—the general efficiency with which individuals carry out basic cognitive operations (Salthouse, 1996)—may therefore play a critical role in production. Prior work has shown that individual differences in processing speed are associated with speech onset latencies in picture-naming tasks (Hintz, Jongman, et al., 2020), suggesting that faster general processing facilitates lexical retrieval. However, it remains unclear whether processing speed similarly supports the planning and production of larger linguistic units, such as phrases and sentences, which place greater demands on the coordination of multiple processes, particularly when considered alongside other domain-general and linguistic predictors.

In addition to domain-general cognitive abilities, language production is shaped by speakers’ linguistic experience. Extensive experience in using language is thought to result in higher-quality lexical representations, which in turn support rapid lexical access (Diependaele et al., 2013; Jongman et al., 2021; Mainz et al., 2017). Linguistic experience, particularly with written language, has also been linked to more efficient processing of syntactic structures, including less frequent constructions such as passives (Favier et al., 2021). These findings suggest that linguistic experience may facilitate both lexical retrieval and structural assembly, and that it is therefore an important factor to consider alongside domain-general skills.

Individual differences approaches provide a powerful framework for investigating how these various factors jointly support language production in general and in lexical retrieval and structural assembly in particular. Rather than focusing on average performance, individual differences research leverages structured variability in behavior to identify shared and distinct sources of variance across tasks. When combined with latent-variable methods, this approach allows researchers to reduce task-specific noise and to characterize underlying constructs more precisely (Borsboom et al., 2003). Examining multiple production tasks alongside multiple domain-general and experiential predictors can thus help to clarify which resources are shared across components of production and which are more selectively involved, thereby informing models of the production system (Kidd et al., 2018; McQueen & Meyer, 2019).

## The present study

The present study adopted such a holistic individual differences approach to investigate how lexical retrieval and structural assembly relate to domain-general skills and linguistic experience. Our participants completed three tightly controlled, timed language production tasks. To probe lexical retrieval at the word level, participants completed a picture naming task. To probe structural assembly at the phrase level, participants generated noun phrases of increasing complexity. To probe structural assembly at the sentence level, participants described transitive scenes designed to elicit active- and passive-voice sentences. In all three tasks, production was elicited by pictorial cues. Importantly, the phrase and sentence production tasks were designed to emphasize structural assembly while minimizing lexical retrieval demands, by using a restricted set of high-frequency items in the phrase task and by providing the relevant lexical material prior to each sentence production trial. Although these tasks do not capture the full breadth of naturalistic language production, their high degree of experimental control allows for targeted assessment of core production processes and is well suited to psychometric investigation.

For each production task, we extracted participants’ speech onset latencies and speech durations. Speech onset latencies were taken to index early planning processes prior to articulation, including lexical retrieval and initial syntactic planning. Speech durations were taken to reflect planning and execution processes that unfold after speech initiation, capturing downstream planning and execution demands that arise as utterances are incrementally produced. Participants also completed a battery of tests assessing working memory, processing speed, and linguistic experience. Because individual performance across cognitive tasks is influenced by general ability, we additionally included a measure of nonverbal reasoning, which is strongly correlated with general cognitive ability (Raven et al., 1998), in order to dissociate its contribution from those of the other predictors.

Using linear mixed-effects modeling, we examined how working memory, processing speed, linguistic experience, and nonverbal reasoning predicted both speech onset latencies and speech durations across the three production tasks. We hypothesized that enhanced linguistic experience would be associated with faster production across tasks, reflected in shorter onset latencies and shorter speech durations, consistent with its role in supporting both lexical retrieval and structural assembly. We further hypothesized that working memory would be selectively related to tasks requiring the production of larger linguistic units—namely, phrase and sentence production—by modulating how planning is distributed across time. Specifically, individuals with higher working memory capacity might be expected to engage in more extensive advance planning or, alternatively, to flexibly allocate planning between pre- and postonset phases. As a consequence of the latter possibility, higher working memory capacity was predicted to be associated with reduced speech durations in these tasks. If individuals engage in extensive advance planning, higher working memory capacity should be associated with longer speech onset latencies. Finally, based on prior findings and the central role of processing speed in cognitive performance more generally (Hintz, Jongman, et al., 2020; Salthouse, 1996), we expected processing speed to be associated with production performance across all three tasks, reflected in better processing skills associated with both earlier speech initiation and more efficient utterance completion. Nonverbal reasoning was included as a control variable, and no task-specific predictions were made regarding its effects.

## Methods

### Participants

The present study is based on data taken from a larger research program on individual differences in language skills, carried out at the Max Planck Institute for Psycholinguistics (Nijmegen, The Netherlands). Specifically, the participants completed the Individual Differences in Language Skills (IDLaS-NL) test battery (Hintz et al., 2025), which consists of 31 behavioral tests that measure individual differences in linguistic experience, general cognitive skills and linguistic processing skills. IDLaS-NL was validated in 748 participants tested between 2020 and 2022. While 579 of these participants completed the tests at home via the internet using their own computers, 169 participants completed them at the Max Planck Institute for Psycholinguistics using computers optimized for experimentation. The data from the latter group were taken for the present analysis.

Eighty-five of the participants were women, 82 were men, and two identified as other; the mean age was 22.38 years (*SD* = 2.76, range: 18–30 years). At the time of testing, most participants were enrolled in a study program either at a university or a university of applied sciences. All participants were native speakers of Dutch. All participants gave written informed consent to take part in the study and were paid for participation. Permission to conduct the study had been provided by the Ethics Board of the Social Sciences Faculty of Radboud University.

### General procedure

The participants signed up for a study that consisted of four test sessions of about one hour each. They completed the first two sessions on one day and the remaining two sessions on another day, approximately one week later. The tests were either implemented in Presentation© (Version 20.0, www.neurobs.com) and run “locally” on the laptops or were implemented as a web application in “Frinex” (FRamework for INteractive EXperiments, an environment developed by the technical group at the Max Planck Institute for Psycholinguistics; Monen et al., 2025) and run online in the laptops’ web browser (Chrome, Version 75.0.3770.142). Specifically, all chronometric tests where exact timing was critical were run in Presentation, while the rest was implemented in Frinex.

### Overview of tests

We included the data from 17 behavioral tests in the present analysis (see Table 1 for an overview, featuring short task descriptions and the dependent variable[s] extracted from each test). The three main tests measured word-level (picture naming), phrase-level, and sentence-level production, respectively. The remaining 15 tests measured participants’ linguistic experience (*n* = 6), WM (*n* = 2), processing speed (*n* = 5), and nonverbal reasoning (*n* = 1). As is common practice in individual differences research, the order of tests and the order of trials within each test were held constant across participants to ensure that any procedural or order-related influences were consistent for all participants and therefore not confounded with the individual differences of interest (Miyake et al., 2000).

**Table 1:** Overview of tests, including references, task instructions, dependent variable, loadings to and explained variance in the principal component analyses

Construct	Test	Description	Dependent variable
Production tests			
Word-level production	*Picture naming* (Hintz et al., 2025; Hintz, Jongman, et al., 2020)	Participants name pictures, whose names vary in word frequency, as quickly as possible.	Onset latency & duration
Phrase-level production	*Phrase generation* (Hintz et al., 2025)	Participants are familiarized with 16 common objects. These objects are presented in noun/adjectival phrases of increasing difficulty, which participants are instructed to name as quickly as possible.	Onset latency & duration
Sentence-level production	*Sentence generation* (Menenti et al., 2011)	Participants describe scenes depicting transitive actions (intransitives serve as fillers) as quickly as possible using prespecified verbal material. Color-coding of the characters in the scenes coerces the production of active and passive sentences.	Onset latency & duration
Predictors			
Linguistic experience	*Peabody Picture Vocabulary Test* (Dunn & Dunn, 1997; Schlichting, 2005)	Participants hear spoken words varying in difficulty and select the matching picture among four alternatives.	Last item nr. minus nr. errors
	*Antonym production* (Mainz et al., 2017)	Participants hear a spoken word and are instructed to produce its antonym.	Accuracy
	*Spelling test* (Hintz et al., 2025)	Participants identify incorrectly spelled words in a list of 60 words (half of which are spelled incorrectly).	Hits minus false alarms
	*Dutch Author Recognition Test* (Brysbaert et al., 2020)	Participants identify fictional writers in a list of 132 names (90 are writers).	Hits minus false alarms
	*Idiom recognition* (Hubers et al., 2019; Tilmatine et al., 2021)	Participants select the correct meaning for an idiomatic expression among four alternatives.	Accuracy
	*Prescriptive grammar* (Favier & Huettig, 2021; Hubers et al., 2016)	Participants carry out grammaticality judgements on spoken sentences featuring morpho-syntactic constructions known to be difficult for adult speakers of Dutch.	Accuracy
Processing speed	*Auditory simple RT test* (Hintz et al., 2024, 2025)	Participants respond as quickly as possible to the presentation of a beep by pressing the space bar.	Mean RT
	*Auditory choice RT test* (Hintz et al., 2024, 2025	Participants respond as quickly as possible to the presentation of one of two beeps (high or low) by pressing one of two buttons.	Mean RT
	*Letter comparison* (Huettig & Janse, 2016; Salthouse, 1996)	Participants indicate as quickly as possible whether two letter strings are identical by pressing one of two buttons.	Mean RT
	*Visual simple RT test* (Deary et al., 2011)	Participants respond as quickly as possible to the presentation of a geometrical shape by pressing the space bar.	Mean RT
	*Visual choice RT test* (Deary et al., 2011)	Participants respond as quickly as possible to the presentation of one of two geometrical shapes by pressing one of two buttons.	Mean RT
Auditory & visual working memory	*Digit span forward* (Wechsler, 2004)	Participants are instructed to recall sequences of digits in the order they were encountered (forward version) or in reversed order (backward version).	Nr. correct items (forward run)
	*Digit span backward* (Wechsler, 2004)		Nr. correct items (backward run)
	*Corsi block clicking forward* (Kessels et al., 2008)	Participants are instructed to recall sequences of identical spatially separated blocks by clicking on them in the order they were encountered (forward version) and in the reversed order (backward version).	Nr. correct items (forward run)
	*Corsi block clicking backward* (Kessels et al., 2008)		Nr. correct items (backward run)
Nonverbal reasoning	*Raven’s Advanced Progressive Matrices* (Raven et al., 1998)	Participants indicate which of four possible shapes completes a matrix of geometric patterns.	Accuracy

## Test descriptions

### **Picture naming**

To test participants’ word-production skills, we administered a picture-naming test. In this test, participants were shown photographs of common objects and were asked to name these as quickly as possible (Hintz, Jongman, et al., 2020). The test materials consisted of 40 photographs taken from de Groot et al. (2016) or retrieved online via a search engine. The object names varied substantially in lexical frequency (*M* Zipf frequency = 3.85, *SD* = 0.86, range: 2.04–5.39; as retrieved from the Subtlex Corpus; Keuleers & Brysbaert, 2010). Prevalence norms (Keuleers et al., 2015) indicated that the object names were likely to be known by all participants (*M* prevalence 99.6%, *SD* = 0.4, range: 97.7–100). The average number of phonological neighbors (sum of additions, substitutions, deletions of segments) of the object names was 4.03 (*SD* = 4.10, range: 0–18; as retrieved from CLEARPOND; Marian et al., 2012). Four additional photographs were used as practice trials. All pictures were scaled to 300 × 300 pixels.

The test began with the presentation of the four practice items. On each trial, participants first saw a fixation cross in the center of the screen, which was shown for 800 ms. Then, the target picture was shown for 3 s. After an intertrial interval of one second, the next trial began. Participants’ utterances were recorded. Naming accuracy as well as word onsets and offsets were coded offline using the Praat software (Boersma & Weenink, 2021). The performance indicators were speech onset latency and speech duration for correctly named non-practice trials. In addition to incorrect picture names, self-corrections, hesitations, diminutives, and plural forms were counted as incorrect.

### **Phrase generation**

In the phrase generation test, participants produced noun phrases of varying complexities by describing one or multiple objects presented in a display. All displays referred to a small set of objects, which minimized between-item variability due to differences in ease of lexical access.

The participants were first familiarized with a set of 16 pictures of common objects, selected from the database by de Groot et al. (2016). Four of these pictures were used as practice trials. The remaining twelve pictures were experimental items. The object names were monosyllabic and high in word frequency (Keuleers & Brysbaert, 2010; *M* Zipf frequency = 4.57, *SD* = 0.44, range: 3.85–5.23) and prevalence (Keuleers et al., 2015; *M* prevalence = 1.00, *SD* = 0.00, range: 0.99–1.00). Dutch nouns differ in grammatical gender (common or neuter) and the corresponding definite determiners (de or het). Half of the object names had common gender and half had neuter gender.

The test consisted of four blocks featuring twelve trials each. Block 1 was preceded by four practice trials; Blocks 2 to 4 were each preceded by two practice trials. The twelve objects used in the main part of the test and the four practice objects were repeated in various combinations across the four blocks. The trial structure was the same in all blocks: A fixation cross was shown in the center of the screen for 500 ms and was replaced with the to-be-named object or combination of objects. Participants were instructed to name these as quickly as possible. They were instructed to press the space bar after they were done speaking.

In Block 1, participants were asked to name single objects (e.g., “hond”–dog). In Block 2, they named pairs of objects shown next to each other in noun phrase conjunctions such as “aap en neus” (monkey and nose). In Block 3, they either saw two or three identical objects and named them in phrases such as “drie honden” (*three dogs*), or they saw a single object, in blue or yellow and had to produce an adjective–noun phrase, such as “blauwe hond.” We opted for the color adjectives *blue* (“blauw”) and *yellow* (“geel”) and the two numerals (*two*, “twee”; *three*, “drie”) as in Dutch these words are perceptually and phonologically distinct from each other. Finally, Block 4 required participants to name two or three objects appearing in one of the two colors in complex adjective noun phrases (e.g., “twee blauwe honden”; *two blue dogs*).

Participants’ responses were recorded, starting with the presentation of the visual stimuli. The recording was terminated one second after participants’ button press or timed out after ten seconds (in case participants forgot to press the button). Speech was coded offline using Praat software (Boersma & Weenink, 2021). The performance indicators were speech onset latency and speech duration for correct trials on Blocks 2–4. Since in Block 1, participants named objects in isolation, we excluded these trials from the present analysis to avoid overlap with the picture naming task.

### **Sentence generation**

The sentence generation test was used to assess participants’ ability to formulate transitive sentences within a structured task. The participants described photographs of two actors, a woman and a man, taking part in a transitive action. Using Adobe Photoshop, the two actors were colored in in yellow and blue and participants were instructed to name the yellow character first in each sentence. The color-coding thus coerced the production of active sentences (when the agent was colored in yellow and the patient in blue; e.g., “The man kisses the woman”; Dutch: “De man kust de vrouw”) and passive sentences (when the patient was colored in yellow and the agent in blue; “The woman is being kissed by the man”; Dutch: “De vrouw wordt door de man gekust”). The actors’ color-coding and their position in the photographs (left or right) were counterbalanced.

Forty photographs featuring transitive actions (e.g., X sees Y, X follows Y) were taken from Menenti et al. (2011). To reduce the likelihood of “self-priming,” the tendency to reuse a syntactic structure that was recently produced (Jacobs et al., 2019), the 40 transitive trials were interspersed with 40 trials where participants produced intransitive sentences (e.g., X lies). Specifically, between two transitive trials, an intransitive filler trial was placed. On these trials, only one agent was present in the photograph, which was colored in yellow. The transitive and intransitive verbs were high in word frequency (*M* Zipf frequency = 4.06, *SD* = 0.86), range: 2.70–5.78) and word prevalence (*M* word prevalence = 1.00, *SD* = 0.01, range:.0.92–1.00; Keuleers et al., 2015).

In line with the general tendency for preferring active- over passive-voice sentences in production, our pilot study (Hintz, Dijkhuis, et al., 2020) had shown that the passive trials yielded many incorrect responses (e.g., participants producing active rather than passive sentences). Since the dependent variable for the present test was speed based, it was critical to obtain comparable numbers of correct trials for both active- and passive-voice sentences. We therefore presented participants with an auditory sentence before each trial, which featured the same voice as that of the subsequent target production (active, passive, intransitive), but used a different verb. To reduce the likelihood of participants developing strategies, agent and patient gender differed between prime and target sentences on half of the trials. Recordings of the prime sentences were made by a male native speaker of Dutch. The sentences were on average 2,120 ms long (*SD* = 274, range: 1,670–2,886).

Each trial started with the presentation of a fixation cross for 200 ms, which was followed by the playback of the spoken sentence. Next, the verb (infinitive form) to be used in the target production was presented for 200 ms in the center of the screen, which was followed by the presentation of the photograph. The participants were instructed to describe the photograph using the verb provided before the photograph and referring to the actors as “woman” and “man,” naming the yellow-colored person first and the blue-colored person second. They were instructed to push the spacebar after producing a sentence. Participants carried out three practice trials before turning to the main part of the test.

Participants’ responses on nonpractice trials were recorded from the presentation of the photograph and were terminated one second after participants’ button press (or timed out after ten seconds in case participants forgot to press the space bar). Speech was coded offline using Praat software (Boersma & Weenink, 2021). Performance indicators were speech onset latency and speech duration for the 40 transitive trials. Only productions that matched the target structure were considered.

### **Peabody Picture Vocabulary Test**

We used a digitized version of the Dutch Peabody Picture Vocabulary Test (PPVT; Dunn & Dunn, 1997; Schlichting, 2005) to assess receptive vocabulary size. On each trial, participants first previewed four numbered line drawings on their screen. When they were ready, they pressed the space bar on their keyboard to hear the probe word. They had to indicate which of the four pictures best corresponded to the meaning of the spoken word by clicking on it. Participants could listen to the probe word as often as they wanted but had to listen to it at least once before a response was recorded. The test had 17 blocks, each consisting of twelve items of roughly the same difficulty. The test started at block 13 (normed entry block for participants aged between 18 and 35). Based on their performance (four or fewer errors in Block 13), participants’ next block was either more difficult (Block 14) or easier (Block 12) than the entry block. The test terminated when more than eight errors were made within a block that was not the starting block or when a participant reached the last item of the test. The participant’s end score was the difference between the item number of the last test word and the number of errors participants made.

### **Antonym production test**

An open-ended, untimed antonym production test, an adapted version of the test recently developed by Mainz et al. (2017) at the Max Planck Institute for Psycholinguistics, was included to measure productive vocabulary. Participants were provided with a word cue and were instructed to produce its antonym (e.g., cue: hot, antonym: cold). The test consisted of 28 trials (three practice and 25 experimental trials). Before each trial, participants saw a fixation cross for 500 ms, after which the cue word was presented (in written form *and* once in spoken form). Participants provided a spoken response and their answer was recorded. They clicked on a button on the screen to advance to the next trial. The cue words varied in word frequency (Keuleers et al., 2010; *M* = 3.74, *SD* = 0.75, range: 1.70–5.00) and prevalence (Keuleers et al., 2015; *M* = 0.98, *SD* = 0.04, range: 0.85–1.00) and thus in how easily an antonym could be retrieved. Accuracy was coded offline. The performance indicator was the proportion of correct experimental trials.

### **Spelling test**

We designed a test to assess language users’ spelling skills. The test consisted of a list of 60 Dutch words whose spelling adult language users often find difficult. These concern for example the correct use of diaeresis (i.e., “bacteriën”; *bacteria*), the use of double consonants in plural forms (“slimmeriken”; *wise guys*), and use of ei/ij (diphthong [ɛi], i.e., “allerlei”; *all kinds*). Participants were presented with a list of 60 words, in pseudorandom order, divided into three columns of 20 words each. Half of the test words was spelled incorrectly. The ratio of correctly and incorrectly spelled words was not known to the participants. Participants were instructed to use their mouse to tick the boxes next to words they thought were spelled incorrectly. The participant’s performance indicator was the proportion correctly categorized misspelled words minus the proportion incorrectly selected words that were spelled correctly.

### **Dutch Author Recognition Test**

We included a digital version of the Dutch Author Recognition Test (Brysbaert et al., 2020). Participants were presented with a written list of 132 names, divided into three columns of 44 words each. They had to indicate which of the listed persons were authors (e.g., Roald Dahl, Nicci French). Ninety of the listed persons were authors and 42 were nonauthor foils. Authors and nonauthors were listed in pseudorandom order and the ratio of authors/nonauthors was not known to participants. The performance indicator was the proportion of correctly identified authors minus the proportion nonauthors wrongly selected.

### **Idiom recognition test**

Participants’ knowledge of Dutch idiomatic expressions was tested using an idiom recognition test. On each trial, the participants were presented with a Dutch idiom, such as “tussen de regels doorlezen” (*to read between the lines*) and a set of four candidate meanings. They had to select the correct meaning among the set of four candidates.

We selected a subset of ten items from the normative database described by Hubers et al. (2019). In Hubers et al.’s study, Dutch native speakers (on average 26 speakers per item) rated 374 different idiomatic expressions on various dimensions and carried out a multiple-choice meaning recognition test. To vary the item difficulty in our test, the ten selected items ranged between 1.35 and 4.39 on the familiarity rating dimension (1–5 scale) and between 0.15 and 1 in meaning recognition accuracy.

In the present test, an idiom was presented at the top of the screen and four meaning candidates were shown underneath in four quadrants of the screen. The position of the target meaning was varied across trials. Both the idiom and the four candidate meanings could be listened to (by mouse-clicking on a loudspeaker icon) to account for the possibility that some idioms are predominantly encountered in the spoken modality and to reduce the influence of reading skill on recognition performance. The performance indicator was the proportion of correctly recognized idioms.

### **Prescriptive grammar test**

To assess participants’ knowledge of Dutch prescriptive grammar, a recently developed grammaticality judgment test (Favier & Huettig, 2021; Hubers et al., 2016) was used. Participants heard spoken sentences and indicated for each of them whether they thought it was a correct Dutch sentence. The sentences featured five grammatical categories (eight trials per category, 50% correct), which adult native speakers of Dutch often find difficult to use correctly: personal pronouns (“ze,” *they* vs. “hun,” *their*; “ik,”* I* vs. “mij,” *me*), comparatives (“als,” *as* vs. “dan,” *than*), relative pronouns (“die,” *this* vs. “dat,” *that*) and participle formation of complex verbs (e.g., “stofzuigen,” *to vacuum*). Stimuli were recorded in a soundproof booth. Average sentence duration was 4,344 ms (*SD* = 653, range: 3,056–5,901).

Each trial started with the presentation of a fixation cross, which coincided with the playback of the spoken sentence. The fixation cross remained in view for the duration of the sentence. Each sentence was presented only once. Participants could respond during or after the presentation of the sentence by mouse-clicking on the appropriate button on the screen (labelled “correct,” right-hand position and “incorrect,” left-hand position). The mouse-click terminated the trial. The inter-trial interval was 500 ms. The performance indicator was the proportion of correct responses.

### **Auditory reaction time (A-RT) tests**

Two tests tapping response speed to auditory stimuli were used. In both cases, the task was to respond as fast as possible to the onset of an auditory stimulus.

### **Auditory simple reaction time test**

In the simple A-RT test, participants saw a fixation cross in the center of the screen. After an interval varying between one and three seconds, a sine tone (550 Hz, 400 ms) was played. Participants were instructed to press the space bar as soon as they heard the tone, which terminated the trial. After one second, the next trial began. The simple A-RT test consisted of 20 test trials, preceded by eight practice trials. The performance indicator was participants’ mean RT.

### **Auditory choice reaction time test**

In the choice A-RT test, the task was to respond as quickly as possible to each of two auditory stimuli, presented in pseudorandom order, by pressing the associated button. Participants first saw a fixation cross in the center of the screen. After an interval varying between 1 and 3 s, a low or high sine tone (300 and 800 Hz, respectively, both 400 ms) was played. Participants pressed the M key on the keyboard when they heard the high tone, and the Z key when they heard the low tone. The intertrial interval was one second. The choice A-RT test consisted of 40 experimental trials, preceded by 16 practice trials. Average RT was calculated based on correct responses.

### **Letter comparison test**

The letter comparison test was adapted from a paper-and-pencil task developed by Salthouse and colleagues (e.g., Salthouse, 1993). We used the computerized version by Huettig and Janse (2016). The participants’ task was to decide whether or not two strings of letters were identical. The first test block featured pairs of three-letter strings (e.g., TZF) and the second block pairs of six-letter strings (e.g., RNHKTG). Letters were presented in a large, monospaced font (Courier New, font size 70). The space between the letter strings in a pair was 300 pixels. Participants were asked to indicate as quickly and accurately as possible whether the two letter strings were the same or different by pressing the Z-key (“different”) or the M-key (“same”). To start, there were six three-letter practice trials. Each test block consisted of twelve trials containing identical strings and twelve trials with different strings. Each trial started with the presentation of a fixation cross, which stayed on the screen for 600 ms. Subsequently, the two letter strings were presented until a response was made. The next trial began after 1 s. Response speed, as performance indicator, was determined by averaging over participants’ correct responses.

### **Visual reaction time tests (V-RT)**

These tests were the visual counterparts of the A-RT tests. Participants were asked to respond as quickly as possible to the onset of a visual stimulus by pressing the correct button on the button box. Both V-RT tests are based on tests designed by Deary et al. (2011).

### **Visual simple reaction time test**

On each trial of the simple V-RT test, participants first saw a fixation cross in the center of the screen. After an interval varying between one and three seconds, it was replaced by a line drawing of a triangle (200 × 200 pixels, black contours). Participants were instructed to press the spacebar as soon as the triangle appeared. The response terminated the trial. After an intertrial interval of one second, the next trial began. The test consisted of 20 experimental trials, preceded by eight practice trials. The performance indicator was participants’ average RT.

### **Visual choice reaction time test**

On each trial of the choice V-RT test, participants first saw a fixation cross in the center of the screen. After an interval varying between one and three seconds, it was replaced with a line drawing of either a star or a circle (black contours, 200 × 200 pixels). Participants were instructed to press the Z key as fast as possible upon appearance of a star, and the M key upon appearance of a circle. The star and circle appeared equally often throughout the experiment and in pseudorandom order. The test consisted of 40 experimental trials, preceded by 16 practice trials. The performance indicator was participants’ average RT on correct trials.

Before creating average RTs, the data from all processing speed tests underwent a preprocessing pipeline, which—based on data inspection—included removing all RTs faster and slower than 100 and 1,000 ms for the simple RT tests, faster and slower than 200 and 2,000 ms for the choice RT tests and faster and slower than 300 and 3,000 ms for the letter comparison test. The remaining data were log-transformed and inversed such that better performance was associated with larger values. Following preprocessing, each participant retained minimally 80% of the trials.

### **Digit span test (forward and backward)**

We used a computerized version of the digit span test (Wechsler, 2004) to assess auditory working memory. At the beginning of each trial, a fixation cross appeared in the center of the screen. After 2000 ms, the playback of a sequence of spoken digits was initiated while the fixation cross remained in view. Digits were presented at approximately 500 ms intervals. Following auditory playback, a response field appeared at the bottom of the screen and participants were requested to type in the digits in the order they were encountered (forward version) or in the reversed order (backward version). The first two trials of each version featured a two-digit sequence; these were considered practice trials. When at least one of two consecutive trials of the same length was recalled correctly, the sequence was extended by one digit. The test ended when two consecutive trials for a sequence length were responded to incorrectly or when participants reached the end of the test (nine digits in the forward version, eight digits in the backward version). Separate performance indicators were obtained for forward and backward versions, operationalized as the sum of correct responses per version.

### **Corsi block clicking test (forward and backward)**

This test was included to assess visual-spatial short-term memory capacity (Kessels et al., 2008) and formed the counterpart to the auditory digit span test. Participants were presented with nine squares, which were randomly distributed across the screen. Different squares lit up successively at a rate of one square per second. At the end of a sequence, a green frame appeared around the display, prompting participants for a response. The participants were instructed to repeat the sequence by clicking on the respective squares, either by forward repetition or backward reproduction. When clicking on the squares, they briefly lit up in black for 200 ms and then turned blank again. After having reproduced the sequence in forward or backward fashion, participants clicked on a button at the bottom of the screen to proceed to the next trial. They were familiarized with the test, completing two practice trials of two-square sequences. The first experimental trial featured a sequence length of three squares. The sequence length was extended by one square when at least one of two consecutive trials was recalled correctly. The test ended when two consecutive trials for a given sequence length were responded to incorrectly or when participants reached the end of the test (sequence of nine blocks in both versions). The performance indicator was the sum of correct responses on experimental trials in forward and backward versions, respectively.

### **Raven's Advanced Progressive Matrices**

To assess nonverbal reasoning, a computerized version of Raven's Advanced Progressive Matrices (Raven et al., 1998) was used. On each trial, participants indicated which of eight possible shapes completed a matrix of geometric patterns. They selected the shape by clicking on it. Participants could skip items by clicking on a button labelled “Skip”; these items were shown again at the end of the test. When they did not know the answer to a skipped item, participants could click on an “I don’t know” button. There were 36 test items, increasing in difficulty, preceded by six untimed practice items. Participants had 20 minutes to complete the experimental items. Throughout the test, a clock in the right top corner of the screen showed the time remaining. The performance indicator was the proportion of correctly answered experimental items.

Table 2 lists the descriptive statistics for each of the 17 tests. For all measures, there was substantial variability in performance across participants as suggested by the range. Furthermore, the skewness and kurtosis values confirmed that the data were normally distributed: Except for two tests (Dutch Author Recognition Test, Auditory choice reaction time test), all values were below ±1. Similarly, except for the Antonym production and Idiom recognition tests, internal consistency ranged between acceptable and excellent. Before validating the battery in the large sample of 748 participants, it was administered to a sample of 112 participants (Hintz, Dijkhuis, et al., 2020). All participants in this sample completed the battery twice, approximately one month apart, thus providing data to assess test–retest reliability, which is critical for measuring stable person variables (Aldridge et al., 2017). The coefficients from the retest correlations have been added to Table 2. The values ranged between acceptable and excellent, except for both scores extracted from the Corsi block test. Note that in response to the low retest values in Hintz, Dijkhuis, et al. (2020), Hintz et al. (2025) adjusted the task instructions for this test, as confusions concerning the task were a likely cause for the poor initial psychometric properties, including internal consistency and retest reliability.

**Table 2:** Descriptive statistics for all variables

Construct	Test	*N*	Incorrect (%)	Trimmed (%)	Mean (*SD*)	Range	Skewness	Kurtosis	IC	Retest- reliability^a^
Word-level production	Picture naming onset	159	7.20	0	951 (294)	323–2808	1.74	4.20	0.9	0.69
	Picture naming duration	159	7.20	0	515 (122)	133–1504	0.60	1.29	0.98	–
Phrase-level production	Phrase generation onset	165	3.18	0	803 (198)	424–2605	1.98	7.55	0.92	–
	Phrase generation duration	165	3.18	0	869 (252)	263–3421	1.20	3.84	0.98	–
Sentence-level production	Sentence generation onset	153	6.99	0	1192 (458)	460–5135	2.12	8.16	0.97	–
	Sentence generation duration	153	6.99	0	1525 (394)	165–4459	1.30	3.92	0.97	–
Linguistic experience	Peabody Picture Vocabulary Test	169	–	–	175.88 (9.89)	136–198	−0.60	0.94	0.89	0.91
	Antonym production	169	–	–	0.80 (0.07)	0.52–0.96	−0.31	0.62	0.44	0.74
	Spelling test	169	–	–	0.58 (0.17)	0.10–0.93	−0.28	−0.37	0.77	0.85
	Dutch Author Recognition Test	166	–	–	0.26 (0.14)	0.02–0.80	1.05	1.24	0.93	0.95
	Idiom recognition	169	–	–	0.77 (0.11)	0.40–1.0	−0.50	0.28	0.19	0.78
	Prescriptive grammar	169	–	–	0.69 (0.10)	0.425–0.925	0.00	−0.29	0.61	0.86
Processing speed	Auditory simple RT test	168	–	0.77	212 (34)	146–356	−0.31	0.51	0.90	0.59
	Auditory choice RT test	165	3.52	1.65	384 (86)	265–915	−1.10	1.84	0.96	0.76
	Letter comparison	169	6.16	0.71	1061 (186)	687–1680	−0.08	−0.07	0.97	0.83
	Visual simple RT test	169	–	0.27	236 (29)	178–360	−0.67	0.51	0.87	0.58
	Visual choice RT test	168	3.56	0.06	408 (59)	297–624	−0.40	0.23	0.93	0.78
Auditory & visual working memory	Digit span test: forward Digit span test: backward	169 168	– –	– –	8.51 (1.76) 7.79 (2.05)	4–14 1–12	0.29 0.03	−0.66 −0.40	0.71 0.71	0.75 0.70
	Corsi block test: forward Corsi block test: backward	166 168	– –	– –	8.27 (1.93) 7.60 (2.03)	4–13 2–14	0.09 0.23	−0.05 0.41	0.60 0.69	0.39 0.49
Nonverbal reasoning	Raven’s Advanced Progressive Matrices	166	–	–	0.60 (0.15)	0.22–0.94	−0.13	−0.33	0.82	0.87

*Note.* IC = Internal consistency. ^a^Test–retest reliability taken from Hintz, Dijkhuis, et al. (2020)

## Construct validation of IDLaS-NL

IDLaS-NL was designed to measure eight different constructs, including linguistic experience, processing speed, WM, nonverbal reasoning, word production, sentence production, word comprehension and sentence comprehension. Hintz et al. (2025) used confirmatory factor analysis to validate the battery’s factor structure. The results showed excellent fit for the linguistic experience factor, with all loadings stronger than .63 (except for idiom recognition). Similarly, the analysis showed that WM was well captured by digit span and Corsi block tests, which were set to load on subordinate, domain-specific auditory WM and visual WM tests, respectively. Both subordinate factors consistently loaded on the superordinate, domain-general WM factor. The analyses further showed that processing speed is best captured by two independent correlated factors—one comprising both auditory tests (ASRT, ACRT) and one comprising the three visual tests (VSRT, VCRT, letter comparison). Finally, concerning picture naming, phrase and sentence generation tests, the confirmatory factor analysis suggested that these tests are best subsumed in a common factor labelled “picture description” rather than in separate factors for word production and sentence production. In hindsight, this grouping can be explained with the nature of the task and the fact that in all three tests timed spoken responses were cued by pictorial input (Hintz et al., 2025, for further discussion). For the present analysis, this was less of a concern as we did not aim to compare performance on these tasks to other production tasks not involving visual input (e.g., verbal fluency). In fact, our goal was to keep the trial structure across word, phrase, and sentence production tasks as similar as possible.

**Fig. 1:**
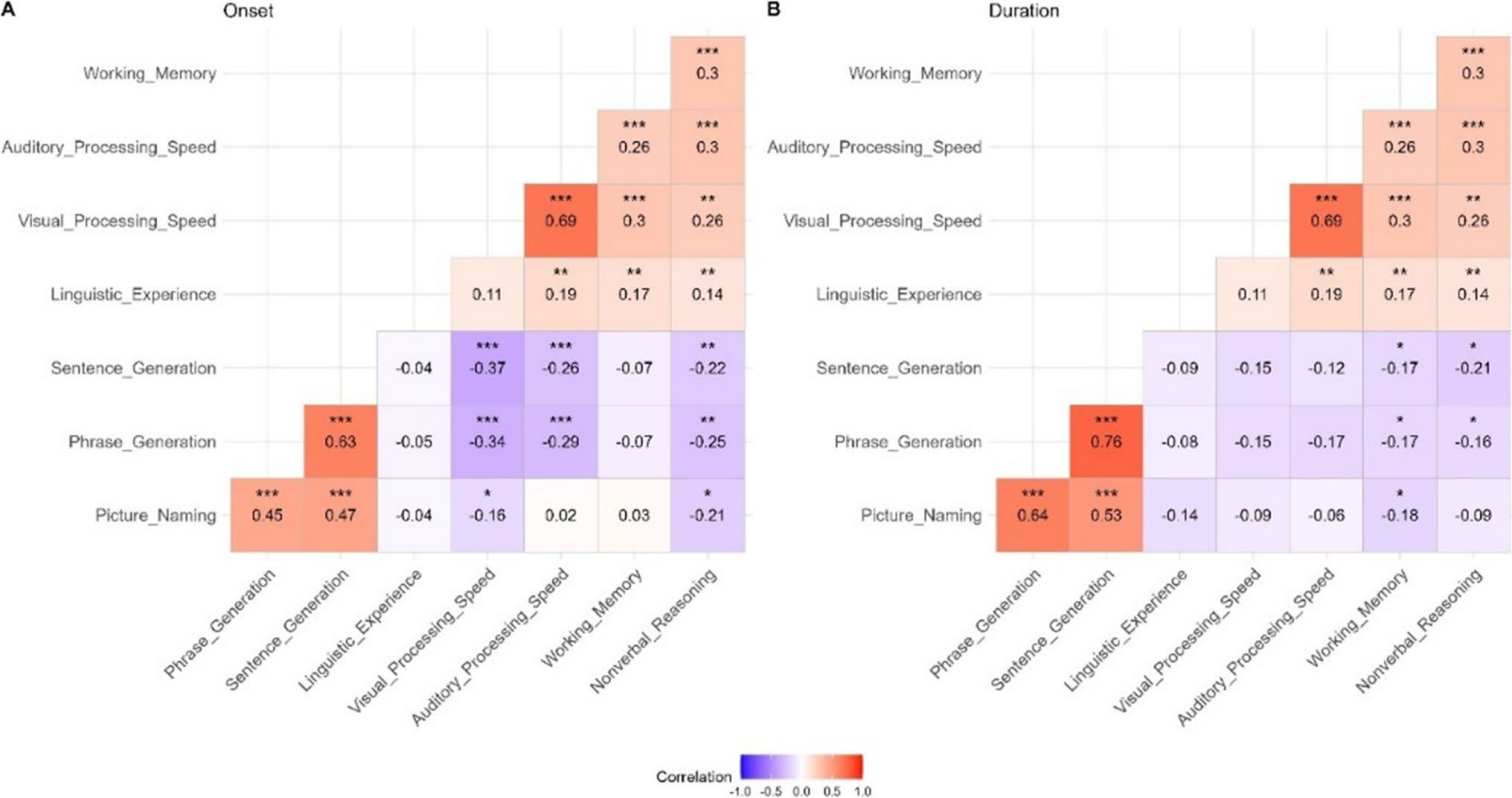
**A** Pearson’s correlation between average speech onset latencies in picture naming, phrase, and sentence generation tasks and all predictor variables. **B** Pearson’s correlation between average speech durations in picture naming, phrase, and sentence generation tasks and all predictor variables. Warmer (i.e., red) colors reflect increasingly more positive correlations and colder (i.e., blue) colors reflect increasingly more negative correlations

To test the explanatory power of the predictors on each language production task, we used the factor scores as obtained in the analyses summarized above. Since the confirmatory factor analysis for processing speed yielded separate scores for auditory and visual processing speed, which were strongly correlated at *r* =.69 (Fig. 1), we ran two models: one with auditory processing speed (APS) and all other predictor variables and one with visual processing speed (VPS) and all other variables. Since nonverbal reasoning was assessed with only one test, we used the score obtained from Raven’s Advanced Progressive Matrices. Figures S1 and S2 in the Appendix show the correlations between the scores (including participants’ average onset latency and speech duration in the three production tasks) from all 17 tests.

## Linear mixed-effects modeling

We fit linear mixed-effects models with onset latency and duration of response as dependent variables, respectively, using the *lme4* (Bates, Mächler, et al., 2015a, 2015b) and *afex* (Singmann, et al., 2024) packages in R (Version 4.5.2.). We conducted variance inflation factor (VIF) analyses to check for high degrees of multicollinearity before proceeding with the planned analysis. Based on Craney and Surles (2002), variables with VIF values above 5 were removed from the analysis.

Motivated by Lo and Andrews (2015), we performed the analysis on non-transformed dependent variables (i.e., onset and duration latency in ms). To accommodate the nonnormal distribution of speed-based dependent variables, we fit three models with “identity link” using Gaussian, gamma, and inverse Gaussian distributions, and two models with “inverse link” using gamma and inverse Gaussian distributions. All were “maximal models” (Barr et al., 2013) without correlation parameters in the random effects structure. We then used the Akaike information criterion (Sakamoto et al., 1986) along with diagnostic plots to determine which distribution and link fit the distribution of our data the best.

To find the most parsimonious model (see Matuschek et al., 2017), we performed a singular value decomposition on the covariance matrix of the maximal model using PCA (Bates, Kliegl, et al., 2015). We removed the random effect with the lowest variance and used likelihood ratio tests (LRTs) to compare the models with each other. The correlation parameters in the random effects structure (both for participants and items) were constrained to zero before modeling to avoid convergence problems. However, after the most parsimonious model was determined, we estimated the correlation parameters for this model. LRT was used to assess if adding the correlation parameters would make any significant difference in the model fit.

Our maximal models for both onset and duration analyses consisted of the following predictors: Task as fixed factor (with three levels: picture naming, phrase generation, and sentence generation), which interacted with (1) linguistic experience, (2) WM, (3) nonverbal reasoning, and (4) VPS, or (5) APS. The continuous predictors were scaled and centered. Task was deviation coded (Brehm & Alday, 2022). The three levels of task (phrase generation, picture naming, and sentence generation) were coded with two contrasts (1, 0, −1) and (0, 1, −1). The random effects in the model included: item intercept with by-item random slopes for linguistic experience, VPS, APS, WM and nonverbal reasoning and participant intercept with by-participant random slopes for task. After the most parsimonious random-effects structure had been determined, we removed observations with absolute standardized residuals exceeding 2.5 standard deviations following a recommendation by Baayen and Milin (2010). The removal of such observations allowed us to assess if the results were influenced by outlier residuals or not. To determine which predictor variables and interactions were significant in the final model, conditional *F* tests were used. *F* tests provide more reliable results than LRT (Halekoh & Højsgaard, 2014; Luke, 2017; Pinheiro & Bates, 2000). Kenward–Roger approximations were used to create denominator degrees of freedom (Kuznetsova et al., 2017; Luke, 2017). Model diagnostics were assessed by checking the normality of residuals and homogeneity of variance for all models using the performance package (Lüdecke et al., 2021).

## Model selection

Trials without a response (13.37%) were removed before the analysis. The results of the VIF analysis showed that multicollinearity was not an issue—all VIFs were below 5. Based on AIC and diagnostic plots, the distribution that fit the onset latency data best was “inverse Gaussian distribution” with “inverse link.” For speech duration, the best distribution was “Gamma distribution” with “identity link.” The model structure that fit both onset latency and speech duration data the best was the maximal model with the structure below. Models with zero correlation parameters for Item performed better than nonzero parameters:

$$\begin{array}{c}Onset/Duration\;latency\sim Task\ast\left(Linguistic\;experience+VPS\;or\;APS+WM+Nonverbal\;reasoning\right)+\\(1+Linguistic\;experience+VPS\;or\;APS+WM+Nonverbal\;reasoning\parallel Item)+(1+Task\mid Participant)\end{array}$$

## Results

### Onset latency analysis

The heatmap in Fig. 1A shows that onset latencies on the three production tasks were significantly correlated. As one would expect, phrase and sentence generation, capitalizing on structural assembly, were more strongly correlated (*r* = .63) than picture naming and phrase/sentence generation (both *r* =.47). Interestingly, neither the linguistic experience nor the WM factor score were significantly correlated with onset latency. Except for APS and picture naming, onset latencies on all language production tasks were negatively correlated with both types of processing speed (i.e., better processing speed abilities associated with shorter onset latencies). Nonverbal reasoning, included as a control variable, correlated negatively with all production tasks (i.e., better reasoning associated with shorter onset latencies) and positively with the other predictor variables.

Table 3 lists the effects of the predictor variables and their interaction with task in both models (Model 1 with APS and Model 2 with VPS). Since task was deviation-coded, we ran separate comparisons between the three levels, which all turned out to be statistically significant: Onset latencies on the phrase generation task were shorter than on the picture naming and sentence generation tasks and onset latencies on the picture naming task were shorter than on the sentence generation task.

**Table 3:** Results of the linear mixed-effects model analyses on speech onset latencies

	Model 1				Model 2	
Predictors	Estimate	95% CI	*p*	Estimate	95% CI	*p*
Intercept*	−1.15	−1.16, −1.14	<0.001*	−1.15	−1.17, −1.14	<0.001*
NVR	−0.02	−0.03, −0.01	<0.001*	−0.02	−0.3, −0.01	<0.001*
LE	−0.01	−0.02, −0.006	<0.01*	−0.02	−0.03, −0.01	<0.001*
PS*	−0.02	−0.03, −0.01	<0.001*	−0.04	−0.05, −0.03	<0.001*
WM	0.007	−0.004, 0.01	0.22	0.01	0.001, 0.02	<0.05*
PG × NVR	−0.0002	−0.01, 0.01	0.96	−0.003	−0.01, 0.009	0.63
PN × NVR	−0.003	−0.01, 0.009	0.58	−0.0008	−0.01, 0.01	0.90
PG × LE	0.005	−0.007, 0.01	0.40	0.001	−0.01, 0.01	0.76
PN × LE	−0.01	−0.02, −0.004	<0.05*	−0.006	−0.01, 0.006	0.32
PG × PS	−0.01	−0.02, −0.002	<0.05*	0.0004	−0.01, 0.01	0.94
PN × PS	0.03	0.02, 0.04	<0.001*	0.02	0.009, 0.03	<0.001*
PG × WM	−0.0008	−0.01, 0.01	0.88	−0.004	−0.01, 0.008	0.52
PN × WM	0.007	−0.005, 0.02	0.25	0.008	−0.005, 0.02	0.22
Random effects	Variance	*SD*		Variance	*SD*	
Participant_Intcpt PG PN	0.003 0.003 0.004	0.06 0.06 0.06		0.003 0.003 0.004	0.06 0.06 0.06	
Item_Intcpt	0.003	0.05		0.003	0.05	
NVR	0.0002	0.01		0.0002	0.01	
LE	0.0003	0.01		0.0003	0.01	
PS	0.0002	0.01		0.0002	0.01	
WM	0.0002	0.01		0.0002	0.01	
Residual	0.00004	0.007		0.00004	0.007	

*NVR* nonverbal reasoning, *LE *linguistic experience, *PS* processing speed, *WM* working memory, *PG* phrase generation, *PN* picture naming. Intercept* is grand mean in the fixed effects.

PS* in Model 1 is auditory processing speed. PS* in Model 2 is visual processing speed. CI was calculated based on Wald method. Model estimates are based on inverse Gaussian distribution with an inverse link

Across both models, we additionally observed simple effects of linguistic experience, processing speed, nonverbal reasoning and WM. Enhanced linguistic experience, better processing speed and nonverbal reasoning skills were associated with shorter onset latencies. The effect of WM, albeit a small one (only observed in Model 2), suggested that better WM skills were associated with longer onset latencies.

**Fig. 2:**
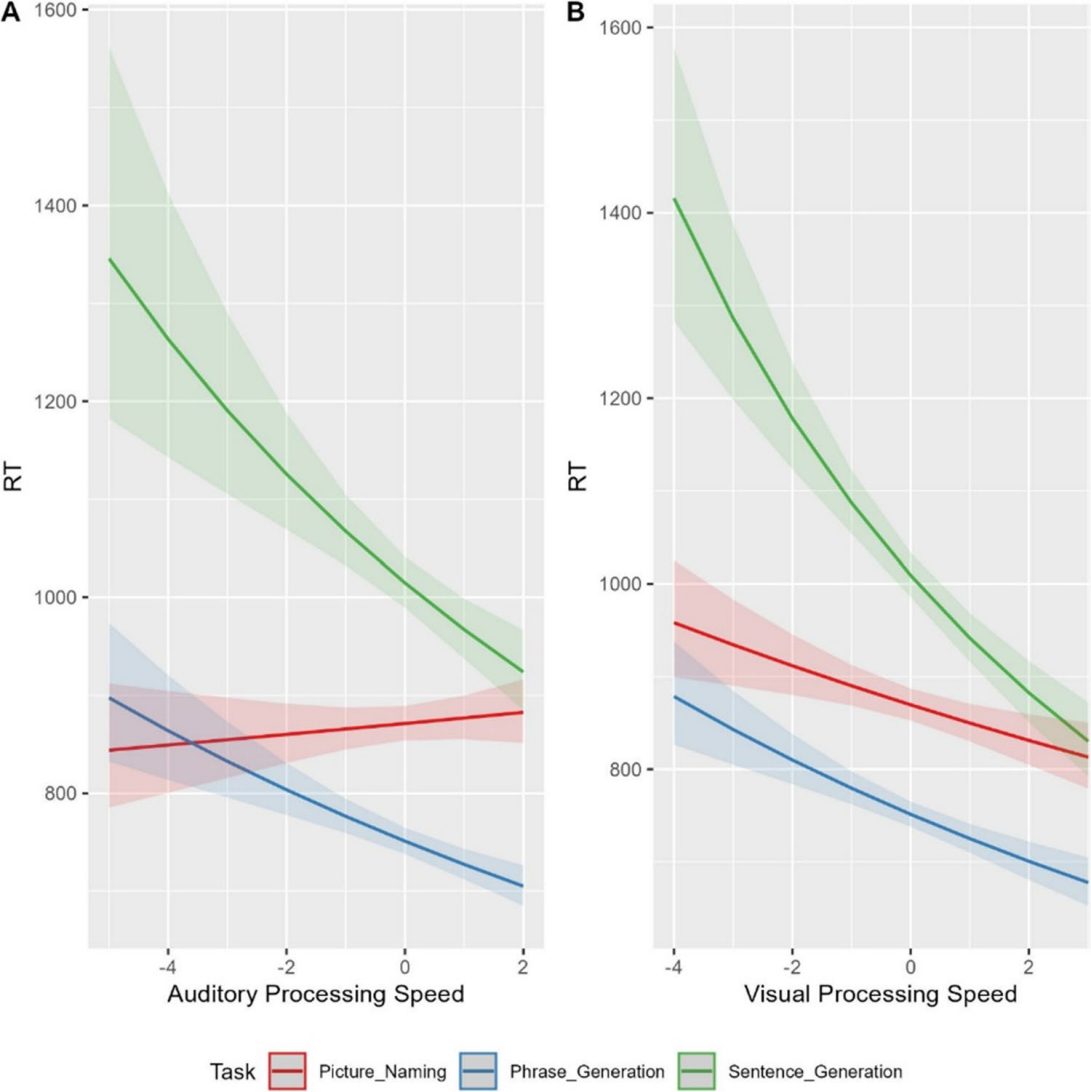
Task × Processing speed interactions in the speech onset latency analyses. (Color figure online)

For both APS and VPS, we additionally observed interaction effects across both models (visualized in Fig. 2). In line with the correlations plotted in Figure 1A, the interactions suggest that APS modulated phrase and sentence generation onset latencies but *not* picture naming (the effects were more pronounced in the model including APS). This suggests that the simple effect of APS was driven by its involvement in phrase and sentence generation.

**Fig. 3:**
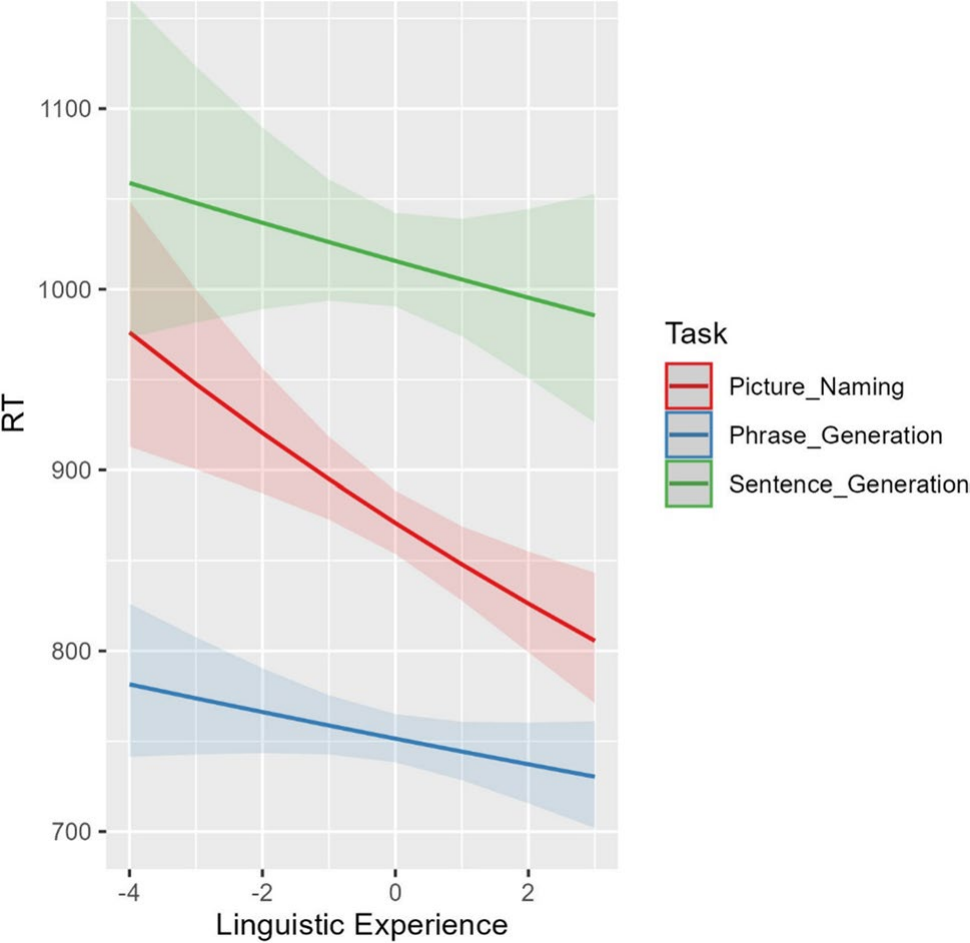
Task × Linguistic Experience interaction in the speech onset latency analysis. (Color figure online)

Finally, in the model containing APS, we observed a complementary effect, namely an interaction between task and linguistic experience (visualized in Fig. 3) suggesting that enhanced linguistic experience benefited picture naming onset latencies more than it benefited phrase and sentence generation.

Interestingly, although the zero-order correlations between working memory and onset latency were small and nonsignificant (Fig. 1), working memory emerged as a positive predictor of onset latency in the mixed-effects model when processing speed and linguistic experience were included as covariates (Table 3). This pattern suggests that once variance shared between working memory, processing speed, and linguistic experience is accounted for, the remaining variance in working memory is associated with longer initiation times.

## Speech duration analysis

The heatmap in Figure 1B shows that the three speech duration measures were significantly correlated (all above *r* = .53), with sentence generation and phrase generation showing the strongest association (*r* = .76). WM, which showed no significant association with language production in the onset latency analysis, correlated significantly with speech duration in all three production tasks (higher WM capacity associated with shorter duration, *r* = .17). Furthermore, nonverbal reasoning correlated significantly with phrase and sentence but not picture naming speech durations. In the correlation analysis, neither visual nor auditory processing speed were significantly correlated with speech duration in any of the three tasks.

**Fig. 4:**
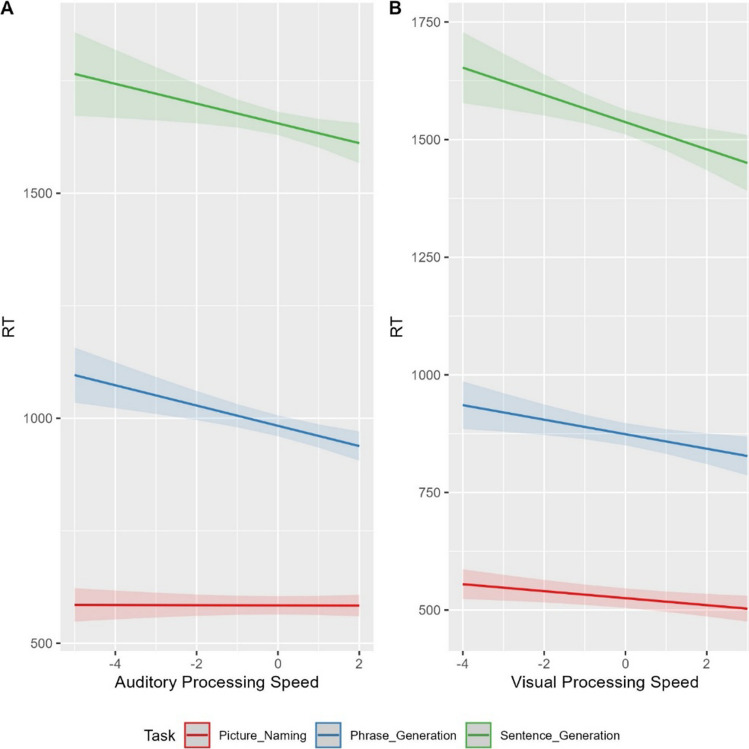
Task × Processing Speed interactions in the speech duration analyses

**Fig. 5:**
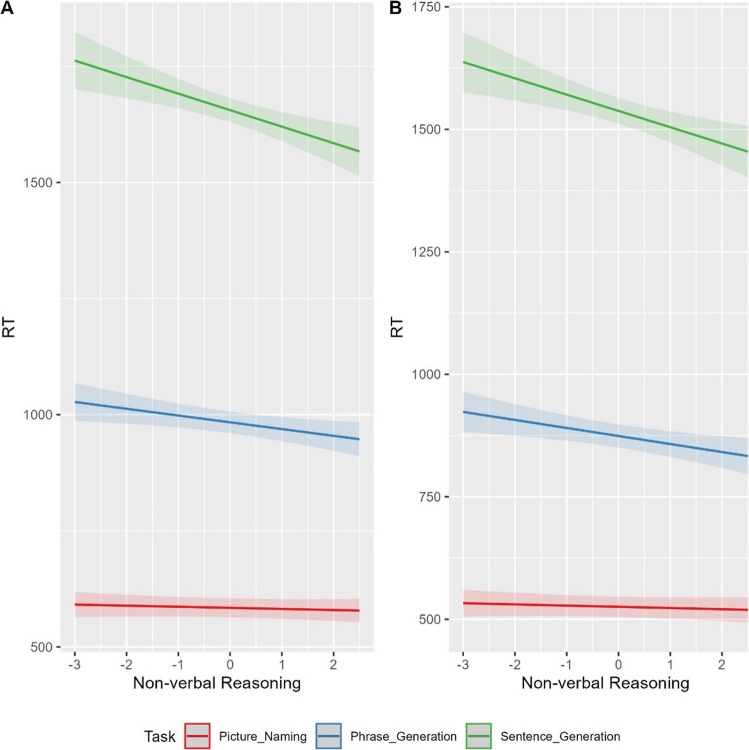
Task × Nonverbal Reasoning interaction in the speech duration analysis. **A** Model containing APS. **B** Model containing VPS

In the mixed model analysis, however, we observed simple effects of processing speed (Table 4) such that shorter speech durations (across all three tasks) were associated with better processing speed skills. However, as in our onset latency analysis, we further observed interactions suggesting that these simple effects were largely driven by specific patterns: Processing speed benefited (i.e., facilitated) structure building (i.e., phrase and sentence generation) more than it benefited picture naming (visualized in Figure 4). In line with the correlation analysis, WM showed a simple effect such that shorter speech durations were associated with higher WM capacity. The analysis also revealed an interaction between nonverbal reasoning and picture naming, suggesting that nonverbal reasoning benefited phrase and sentence generation more (i.e., larger Raven’s scores associated with shorter speech durations) than it benefited picture naming (visualized in Fig. 5). Linguistic experience showed no effects on speech durations.

**Table 4:** Results of the linear mixed-effects model analyses on speech duration

	Model 1				Model 2	
Predictors	Estimate	95% CI	*p*	Estimate	95% CI	*p*
Intercept*	1,074.01	1059.77, 1088.26	<0.001*	978.52	964.06, 992.98	<0.001*
NVR	−17.50	−26.60, −8.39	<0.001*	−17.36	−26.48, −8.25	<0.001*
LE	0.03	−8.77, 8.84	0.99	−5.95	−14.74, 2.82	0.18
PS*	−14.90	−23.95, −5.84	<0.01*	−17.27	−26.29, −8.24	<0.001*
WM	−18.58	−27.67, −9.48	<0.001*	−14.92	−24.19, −5.64	<0.01*
PG × NVR	2.92	−4.60, 10.88	0.44	0.97	−6.46, 8.42	0.79
PN × NVR	15.14	7.12, 23.17	<0.001*	14.90	6.93, 22.88	<0.001*
PG × LE	2.40	−5.04, 9.86	0.52	−0.002	−7.34, 7.34	0.99
PN × LE	−5.90	−13.79, 1.97	0.14	0.15	−7.65, 7.97	0.96
PG × PS	−7.61	−15.15, −0.07	<0.05*	1.83	−5.49, 9.15	0.62
PN × PS	14.67	6.68, 22.67	<0.001*	9.83	1.98, 17.68	<0.05*
PG × WM	0.57	−6.91, 8.07	0.87	−2.03	−9.57, 5.51	0.59
PN × WM	4.23	−3.79, 12.26	0.30	2.34	−5.78, 10.48	0.57
Random effects	Variance	*SD*		Variance	*SD*	
Participant_Intcpt PG PN	2,694.91 1,550.66 1,924.46	51.91 39.37 43.86		2,743.14 1,535.73 1,927.83	52.37 39.18 43.90	
Item_Intcpt	3,985.38	63.12		4,140.34	64.34	
NVR	13.19	3.63		14.79	3.84	
LE	45.01	6.70		47.20	6.87	
PS	24.15	4.91		12.82	3.58	
WM	35.46	5.95		36.17	6.01	
Residual	0.02	0.14		0.01	0.14	

*NVR* nonverbal reasoning, *LE* linguistic experience, *PS* processing speed, *WM* working memory, *PG* phrase generation, *PN* picture naming. Intercept* is grand mean in the fixed effects.

PS* in Model 1 is auditory processing speed. PS* in Model 2 is visual processing speed. CI is calculated based on Wald method. Model estimates are based on gamma distribution with an identity link

## Discussion

To produce well-formed sentences, speakers must coordinate two processes: They need to retrieve lexical representations of the words they intend to produce and they must engage in structural assembly (Bock & Ferreira, 2014; Levelt, 1999; Vigliocco & Hartsuiker, 2002). The present study investigated individual variability in these processes by examining performance on a picture naming task (assumed to tap into lexical retrieval) and in phrase and sentence generation (assumed to tap into structural assembly). We further examined how performance on these three production tasks relates to domain-general skills (working memory, processing speed, nonverbal reasoning) and linguistic experience. In doing so, we moved beyond earlier work and characterized how multiple predictors *jointly* account for variability in different components of production.

Language production beyond the word level is widely assumed to be incremental, allowing speakers to distribute planning across pre- and postonset phases. Capturing this incrementality requires measures that index different stages of the production process. In the present study, we focused on speech onset latencies (the time speakers take before initiating an utterance) and speech durations (the time required to complete the utterance once articulation has begun). These measures provide complementary insights: Onset latencies primarily reflect planning processes that occur prior to speech initiation, whereas speech durations capture planning and execution demands that arise during articulation.

We observed significant differences in onset latencies between the tasks, with shorter onset latencies for phrases than for picture names and shorter onset latencies for picture names than for sentences. That onset latencies for phrases were shorter than for pictures was unexpected and most likely due to the phrase generation task including only a small set of monosyllabic, high-frequency words whereas picture naming included both mono- and disyllabic words, which varied substantially in frequency and therefore took longer to retrieve. Speech durations also differed across tasks, with shortest speech durations for single words, followed by phrases and sentences.

Our analyses revealed that greater linguistic experience, better processing speed, and higher nonverbal reasoning were associated with shorter onset latencies across tasks, while higher working memory capacity was associated with longer onset latencies. For speech durations, better processing speed, working memory, and nonverbal reasoning all led to shorter durations. Importantly, for both measures, interactions showed that processing speed had a greater impact on phrase and sentence generation than on picture naming. Furthermore, while linguistic experience had a stronger effect on shortening onset latencies in picture naming than in phrase and sentence generation, nonverbal reasoning shortened speech durations more in phrase and sentence generation than in picture naming.

In the following sections, we discuss in more detail how each of the predictor variables contributes to language production, beginning with the role of working memory.

### Working memory and the allocation of planning across time

Rather than acting as a simple capacity constraint that uniformly speeds or slows production, working memory appears to modulate how speakers allocate planning across time (see also Swets & Ivanova, 2022; Swets et al., 2014). In the present study, working memory was associated with both speech onset latencies and speech durations, but in opposite directions: higher working memory capacity was related to longer onset latencies, yet to shorter speech durations. This pattern suggests that speakers with greater working memory capacity engage in more extensive advance planning before speech onset, resulting in later initiation but more efficient resolution of planning demands during articulation, as reflected in shorter utterance durations. Conversely, speakers with lower working memory capacity may initiate speech earlier with less advance planning, thereby shifting planning demands to later stages of production.

Importantly, the positive association between working memory and onset latency was observed only when conditioning on other abilities correlated with working memory (i.e., processing speed and linguistic experience). In zero-order terms, working memory was not reliably associated with latency. This suggests that longer onset latencies are not a general property of individuals with higher working memory capacity, but rather emerge from the component of working memory that is independent of processing speed and experience. We therefore interpret the latency effect cautiously, as consistent with—but not on its own conclusive evidence for—greater advance planning in individuals with higher working memory capacity.

### Processing speed affects structural assembly

Processing speed emerged as a robust predictor of production performance across both speech onset latencies and speech durations, with higher processing speed associated with faster initiation and more rapid completion of utterances. Crucially, however, we observed interactions with task for both measures, suggesting that the benefits of processing speed were larger for phrase and sentence generation than for picture naming. This pattern indicates that processing speed plays a particularly important role when production requires the assembly of multiword structures rather than the retrieval of single lexical items. Unlike working memory, which appears to modulate how speech planning is distributed across time, processing speed seems to support the efficient coordination of processes as a function of structural complexity. Producing phrases and sentences affords the rapid integration of lexical and structural information under time pressure, and individuals with higher processing speed may be better able to handle these demands, resulting in both earlier speech initiation and shorter utterance durations.

We note that this account clashes with the data reported in Hintz, Jongman, et al. (2020), where processing speed but not linguistic experience was significantly correlated with picture naming onset latencies. There are some methodological differences between Hintz, Jongman, et al. (2020) and the present study. In the earlier study, linguistic experience was assessed in only one test (Peabody Picture Vocabulary Test). Moreover, rather than differentiating an auditory and a visual factor, processing speed in the earlier study was operationalized by conducting a principal component analysis on the five tests also used in the present study and additionally the digit-symbol substitution test (Wechsler, 2004). Finally, the participants in the earlier study featured a larger variety in educational backgrounds, ranging from vocational job training to university programs. It is unclear how precisely the combination of these differences may have contributed to the divergent patterns of results observed across the two studies. Accordingly, the present results need to be replicated in future research to confirm their robustness.

### Linguistic experience and representational quality

Linguistic experience was associated with overall faster speech initiation, as reflected in shorter onset latencies. Crucially, this effect was qualified by an interaction with task, suggesting that linguistic experience benefited picture naming more than phrase and sentence generation. This pattern suggests that linguistic experience exerts its strongest influence on lexical retrieval, consistent with the idea that extensive exposure to language enhances the quality and accessibility of lexical representations (Diependaele et al., 2013; Hintz et al., 2026; Huettig & Pickering, 2019; Yap et al., 2009). High-quality lexical representations can be retrieved more efficiently, facilitating rapid speech initiation in tasks that place primary demands on word-level processing (e.g., picture naming). Linguistic experience did not affect speech durations, further supporting the interpretation that, at least in the tasks used here, its primary contribution lies in lexical retrieval. Because picture naming minimally engages downstream planning once a word has been retrieved (e.g., Meyer et al., 1998), onset latency constitutes the most informative measure of performance in this task.

### The interplay between domain-general skills and linguistic experience in language production

Taken together, the results suggest that language production is supported by a combination of domain-specific linguistic experience and domain-general skills, each contributing in distinct ways. We interpret our data to suggest that linguistic experience shapes the quality of representations, processing speed supports efficient coordination, and working memory modulates how planning is distributed across time.

One way to interpret these dissociations is by drawing a functional distinction between automatized lexical retrieval and structural assembly. Lexical retrieval, as engaged during picture naming, is a highly practiced and routinized process that draws on entrenched mappings between concepts and word forms in long-term memory. As such, it benefits most from accumulated linguistic experience, which sharpens the strength, accessibility, and specificity of lexical representations. Individuals with richer exposure to language—particularly written language—may retrieve words more quickly, not because of faster processing in general but because of more efficiently organized and accessible lexical networks (Diependaele et al., 2013; Yap et al., 2009).

In contrast, structural assembly, as engaged during phrase and sentence generation, requires the integration of multiple linguistic and conceptual elements into well-formed utterances. This integration process imposes dynamic, time-sensitive demands on the cognitive system. Syntactic frames must be retrieved and instantiated, morpho-syntactic agreements resolved, and sequential ordering planned—all of which are carried out under temporal constraints. These operations are less automatized and more variable across utterances and speakers, leaving greater room for domain-general skills, such as processing speed and working memory, to impact performance.

Although our study cannot directly adjudicate between competing models of language production, the differential engagements of processing speed in structural assembly and of linguistic experience in lexical retrieval challenge accounts that propose tightly coupled interactions between these processes (Pickering & Branigan, 1998; Tomasello, 2000). If lexical retrieval and structural assembly were fully integrated, one might expect processing speed and linguistic experience to influence all production tasks equally. Instead, our results align with accounts proposing some degree of independence between these processes (e.g., Allum & Wheeldon, 2007; Chang et al., 2006; Goldberg, 2005; Konopka, 2012; Smith & Wheeldon, 1999). We propose that future confirmatory research is directed at this hypothesis.

### Limitations and future directions

The present study employed highly controlled production tasks that do not approximate naturalistic, discourse-level language use. This limitation is intentional and reflects a trade-off central to the study’s psychometric aims. By tightly constraining lexical content and syntactic alternatives, the tasks were designed to isolate the core components of production—lexical retrieval and structural assembly—while minimizing extraneous sources of variability. Such control is essential for identifying reliable individual differences and for mapping them onto underlying cognitive abilities. At the same time, it constrains the scope of the conclusions that can be drawn.

Despite our efforts to reduce lexical retrieval demands in phrase and sentence generation, participants necessarily retrieved lexical items in all tasks. However, the absence of processing speed effects in picture naming, which places strong demands on lexical retrieval but minimal demands on structural assembly, suggests that the presence of processing speed effects in phrase and sentence production is not readily explained by lexical access.

That said, a central limitation remains: The observed differences between tasks may reflect not only differences in linguistic processing but also differences in broader task demands. Phrase and sentence generation require participants to maintain task instructions and to coordinate multiple processing steps, all of which likely engage domain-general cognitive resources. Consistent with this possibility, nonverbal reasoning—a widely used proxy for individuals’ general cognitive ability (Raven et al., 1998)—was associated with both shorter speech onset latencies and shorter speech durations, and its effects on speech durations were particularly pronounced for phrase and sentence generation. These findings suggest that at least some of the variance related to task complexity and coordination demands is captured by general ability, rather than by linguistic processing per se. We conjecture that this pattern renders it less likely that the task-dependent effects observed for processing speed can be fully explained by non-linguistic task demands. Our findings instead support the interpretation that processing speed contributes to the rapid integration of lexical and morpho-syntactic information (i.e., structural assembly) under time pressure. However, more research is required to substantiate this conjecture.

The present results provide a baseline characterization of how individual differences in working memory, processing speed, nonverbal reasoning and linguistic experience relate to core production processes assessed in well-controlled tasks. While these tasks do not yield process-pure measures of lexical retrieval and structural assembly, they help delineate the contributions of domain-general skills and linguistic experience to different aspects of production. An important direction for future research is to extend this approach to more complex and less constrained production contexts, including longer utterances, discourse-level planning, and interactive dialogue. Combining individual differences designs with explicit computational or process models of production may be particularly valuable for clarifying how task demands, planning strategies, and linguistic processes jointly shape speech production.

Our findings underscore the value of a holistic individual differences approach. By jointly considering multiple predictors, we were able to identify distinct contributions of working memory, processing speed, nonverbal reasoning, and linguistic experience. Future work adopting similar integrative approaches will be crucial for refining models of language production and for understanding how multiple cognitive components interact during speech planning and execution.

## Supplementary Information

Below is the link to the electronic supplementary material.

Supplementary file1 (DOCX 651 KB)

## Data Availability

The data and analysis code are available on OSF (https://osf.io/h7y2d/?view_only=541f081d7a4942439bbf8ce940768f38). The analysis was not preregistered. The materials can be found in Hintz et al. (2025, *Brain Research,* Online Supplement A).
