# Supplementary material for: Linguistic experience and processing speed differentially affect lexical retrieval and structural assembly during language production
Source: Mem Cognit. 2026 Mar 2;54(6):2165–86. doi: 10.3758/s13421-026-01861-x (PMC13461654; doi:10.3758/s13421-026-01861-x)
Supplement: Supplementary file 1 [file 13421_2026_1861_MOESM1_ESM.docx]

**Appendix**

Figure S1

*Correlation matrix (plotted as a heatmap) for all tests scores and* ***speech onset latencies****.* *Warmer (i.e., red) colors reflect increasingly more positive correlations and colder (i.e., blue) colors reflect increasingly more negative correlations.*

*Note*. Abbreviations: PPVT = Peabody Picture Vocabulary Test, AntonymProd = Antonym production, IdiomRec = Idiom recognition, Spelling = Spelling test, DART = Dutch Author Recognition Test, PresGrammar = Prescriptive grammar test, A.SRT = Auditory simple reaction time test, A.CRT = Auditory choice reaction time test, LetterComp = Letter comparison test, V.SRT = Visual simple reaction time test, V.CRT = Visual choice reaction time test, DS forward = Digit span forward, DS backward = Digit span backward, Corsi forward = Corsi block test forward, Corsi backward = Corsi block test backward. For the correlation analysis, picture naming, phrase and sentence generation scores were operationalized as participants’ average onset latency in each test.

Figure S2

*Correlation matrix (plotted as a heatmap) for all tests scores and* ***speech durations****.*

*Note*. Abbreviations: PPVT = Peabody Picture Vocabulary Test, AntonymProd = Antonym production, IdiomRec = Idiom recognition, Spelling = Spelling test, DART = Dutch Author Recognition Test, PresGrammar = Prescriptive grammar test, A.SRT = Auditory simple reaction time test, A.CRT = Auditory choice reaction time test, LetterComp = Letter comparison test, V.SRT = Visual simple reaction time test, V.CRT = Visual choice reaction time test, DS forward = Digit span forward, DS backward = Digit span backward, Corsi forward = Corsi block test forward, Corsi backward = Corsi block test backward. For the correlation analysis, picture naming, phrase and sentence generation scores were operationalized as participants’ average onset latency in each test.
